# On the Responsible Use of Chatbots in Bioinformatics

**DOI:** 10.1093/gpbjnl/qzae002

**Published:** 2024-01-05

**Authors:** Gangqing Hu, Li Liu, Dong Xu

**Affiliations:** Department of Microbiology, Immunology & Cell Biology, West Virginia University, Morgantown, WV 26506, USA; College of Health Solutions, Arizona State University, Phoenix, AZ 85004, USA; Biodesign Institute, Arizona State University, Tempe, AZ 85281, USA; Department of Electrical Engineer and Computer Science, Christopher S. Bond Life Sciences Center, University of Missouri, Columbia, MO 65211, USA

Large language model (LLM)-based chatbots like Chat Generative Pre-trained Transformer (ChatGPT), equipped with broad biological knowledge [1], have demonstrated an impressive capability for bioinformatics coding [2]. When given well-crafted instructions, these chatbots hold the potential to significantly augment bioinformatics education and research [3,4]. However, opportunities entail both rewards and risks. This commentary explores the challenges of using chatbots in bioinformatics and proposes strategies to manage the associated risks while maximizing the benefits.

Most importantly, the effectiveness of a chatbot’s response largely depends on the quality of the prompts. Crafting effective prompts can be challenging, sometimes leading to frustration or discouragement. Pioneering studies have identified various prompting techniques to guide chatbot responses, including role prompting that assigns a role to the chatbot, few-shot prompting that provides relevant examples, and chatbot self-reflection that improves responses based on task feedbacks [4–6]. Furthermore, bioinformatics data analysis requires domain-specific knowledge. Advanced techniques, such as chain-of-thought prompting [7], which dissects complex reasoning into intermediate steps, enable a more organized understanding of specific bioinformatics analyses. Recent developments in GPT Application Programming Interface (API)-based webservers (*e.g.*, Chatlize.ai) and ChatGPT plugins (*e.g.*, Code Interpreter or Advanced Data Analysis) offer user-friendly platforms for practicing bioinformatics with chatbots (also known as “prompt bioinformatics”). A repository of well-crafted examples for commonly used bioinformatics analyses would be an invaluable resource for the community [4]. These prompting skills, API-based applications, and the repository are helpful resources to mitigate prompt-related stress during this type of new bioinformatics practice.

The next challenge deals with uncertainties in chatbot response. A chatbot might generate different code for the same prompt. While some of these variations are due to ambiguities in prompts or simply a chatbot’s propensity for “hallucination”, others may be valid alternative solutions [4]. However, since a chatbot cannot fully evaluate its own responses, users bear the sole responsibility of assessing and controlling the uncertainty on their own. Prompts shall preferably be designed to aim for reproducible results, but code from the chatbot may have inherent variability in different chat sessions. Although technically one can set the chatbot’s “temperature” to zero to have a fixed result, it may not be the best practice as variable results may contain better solutions. It is therefore recommended to test a prompt multiple times and cross-check different responses to seek the best coding solution or to exclude erroneous results.

While emphasizing the significance of developing prompting skills for effective communication with chatbots, it should not overshadow the importance of developing coding skills. One may consider prompting as a substitute for coding. This misconception can lead to a superficial understanding of coding concepts and inhibit progression into more advanced applications. For instance, reliance on chatbots for debugging may hamper the acquisition of vital troubleshooting skills. Moreover, advancing beyond basic prompting necessitates a strong foundation in algorithm design and bioinformatics domain knowledge. To offset these potential negatives, chatbots should function as supporting tools to supplement traditional education. For example, chatbot-assisted bioinformatics lectures could begin with an in-depth guided discussion of the problem at hand and potential solutions. Interacting with the chatbot aims to enhance students’ comprehension of coding concepts, refine their prompting skills, and foster critical thinking [4]. Assignments could involve exploring similar problems, investigating alternative solutions with the chatbot, and critically assessing its responses. This strategy strikes a balance between artificial intelligence (AI) assistance and traditional instruction, fostering self-reliance for bioinformatics learners.

ChatGPT exhibits impressive performance from recent evaluations on data mining [8], genetics knowledge [9], coding [2,10,11], and bioinformatics figure interpretations [12]. It also demonstrates superior performance in coding to its peers such as Bard [2] and other fine-tune-based language models [10] using bioinformatics-specific benchmarks. However, the chatbot has inherent limitations in performing certain types of bioinformatics tasks. Its knowledge is limited by the cut-off date of its training. Data analysis using tools developed post-training could prove challenging. Although ChatGPT added Bing for real-time internet access, its performance in assisting coding is unstable based on our own tests. Feeding the chatbot instructions and user examples may enhance its performance. When it comes to handling tasks that involve human–computer interactions, such as the use of graphical user interfaces (GUIs) or quantitative analysis of visual elements from figures [12], the chatbot falls short on its own. Using chatbots in new algorithm development demands a high level of domain-specific knowledge. In this process, intensive interactions with the chatbot, involving precise instructions, are expected before achieving a meaningful prototype for future development. Equally challenging will be to generate bioinformatics pipelines through chatbots using existing tools. Such expeditions aim to lay the groundwork for the creation of a bioinformatics chatbot. Such a chatbot is envisioned to address a wide array of data analysis questions, many of which are frequently asked but often out of reach for most biomedical scientists, thus making resources more accessible. Given the fast-paced evolution of the AI field, we anticipate that new tools will emerge to address, at least in part, many of these challenges. For instance, the emerging direction in developing prompting skills specific to image inputs [13] holds promise in addressing the limitations associated with GUI usage and the interpretation of scientific figures [12]. Concurrently, it will be essential to update old prompts, previously optimized for earlier AI models, to sustain or boost their performance with the advent of improved models.

Last but not least, ethical guidelines and regulations for using chatbots in coding are still evolving. While code sharing and reuse are common practices in bioinformatics as part of the open science ethos, proper credit should always be given when using AI-generated code [14]. Additionally, details of the chatbot usage should be documented. This should encompass details such as the chatbot name (including version and settings), prompts used, the code generated, alongside any subsequent human modifications, and reference results. It is also essential to display metrics that measure the robustness of the prompts in replicating the reference results. These records should be made publicly accessible to ensure transparency, reproducibility, and accountability. Furthermore, because bioinformatics analysis often deals with genomic and clinical data, including personally identifiable information, the use of chatbots should adhere to regulations on privacy and data security. In this context, it is recommended to confine the usage of LLMs to secured local networks and thoroughly inspect the source code of the model implementation as a precautionary measure against potential breaches.

In conclusion, the incorporation of chatbots into data analysis represents an emerging and promising avenue in bioinformatics. For responsible and productive use of chatbots in this field, it is crucial to thoughtfully devise strategies to address the emerging challenges such as psychological strain from prompt-related frustration, uncertainties in chatbot’s responses, overreliance on AI, and transparency on ensuring data security. As this nascent technology continues to evolve, we remain optimistic that chatbots will not only enhance educational outcomes for learners, but also boost the productivity of seasoned professionals in the field.

## CRediT author statement

**Gangqing Hu:** Conceptualization, Writing – original draft, Writing – review & editing. **Li Liu:** Writing – review & editing. **Dong Xu:** Conceptualization, Writing – review & editing. All authors have read and approved the final manuscript.

## Competing interests

The authors have declared no competing interests.

## References

[1] Hou W, Ji Z. GeneTuring tests GPT models in genomics. bioRxiv 2023;532238.

[2] Piccolo SR, Denny P, Luxton-Reilly A, Payne SH, Ridge PG. Evaluating a large language model’s ability to solve programming exercises from an introductory bioinformatics course. PLoS Comput Biol 2023;19:e1011511. 37769024 10.1371/journal.pcbi.1011511 PMC10564134

[3] Xu D. ChatGPT opens a new door for bioinformatics. Quant Biol 2023;11:204–6. 37900935 10.15302/J-QB-023-0328 PMC10609615

[4] Shue E, Liu L, Li B, Feng Z, Li X, Hu G. Empowering beginners in bioinformatics with ChatGPT. Quant Biol 2023;11:105–8. 37378043 10.15302/J-QB-023-0327 PMC10299548

[5] Shinn N, Cassano F, Labash B, Gopinath A, Narasimhan K, Yao S. Reflexion: language agents with verbal reinforcement learning. arXiv 2023;2303.11366.

[6] Perkel JM. Six tips for better coding with ChatGPT. Nature 2023;618:422–3. 37277596 10.1038/d41586-023-01833-0

[7] Wei J, Wang X, Schuurmans D, Bosma M, Ihter B, Xia F, et al Chain-of-thought prompting elicits reasoning in large language models. Adv Neural Inf Process Syst 2022;35:24824–37.

[8] Chen Q, Sun H, Liu H, Jiang Y, Ran T, Jin X, et al An extensive benchmark study on biomedical text generation and mining with ChatGPT. Bioinformatics 2023;39:btad557. 37682111 10.1093/bioinformatics/btad557 PMC10562950

[9] Duong D, Solomon BD. Analysis of large-language model versus human performance for genetics questions. Eur J Hum Genet 2024;32:466–8. 37246194 10.1038/s41431-023-01396-8 PMC10999420

[10] Tang X, Qian B, Gao R, Chen J, Chen X, Gerstein M. BioCoder: a benchmark for bioinformatics code generation with contextual pragmatic knowledge. arXiv 2023;2308.16458.

[11] Wang L, Ge X, Liu L, Hu G. Code interpreter for bioinformatics: are we there yet? Ann Biomed Eng 2024;52:754–6. 37482573 10.1007/s10439-023-03324-9

[12] Wang J, Ye Q, Liu L, Guo NL, Hu G. Bioinformatics illustrations decoded by ChatGPT: the good, the bad, and the ugly. bioRxiv 2023;562423.

[13] Yang Z, Li L, Lin K, Wang J, Lin C, Liu Z, et al The dawn of LMMs: preliminary explorations with GPT-4V(ision). arXiv 2023;2309.17421.

[14] Merow C, Serra-Diaz JM, Enquist BJ, Wilson AM. AI chatbots can boost scientific coding. Nat Ecol Evol 2023;7:960–2. 37100951 10.1038/s41559-023-02063-3

